# Estimate the burden of malnutrition among children with cerebral palsy in Sub-Saharan Africa: a systematic review with meta-analysis

**DOI:** 10.1038/s41598-024-55730-1

**Published:** 2024-03-18

**Authors:** Ermias Sisay Chanie, Natnael Moges, Fikadie Dagnew Baye, Gebrehiwot Berie Mekonnen, Mengistu Melak Fekadie, Lakachew Yismaw Bazezew, Denekew Tenaw Anley, Melkamu Aderajew Zemene, Natnael Atnafu Gebeyehu, Getachew Asmare Adella, Gizachew Ambaw Kassie, Misganaw Asmamaw Mengstie, Mohammed Abdu Seid, Endeshaw Chekol Abebe, Molalegn Mesele Gesese, Kirubel Dagnaw Tegegne, Yenealem Solomon Kebede, Berihun Bantie, Sefineh Fenta Feleke, Tadesse Asmamaw Dejenie, Wubet Alebachew Bayih, Amare Kassaw, Anteneh Mengist Dessie, Melkalem Mamuye Azanaw, Sewunt Sisay Chanie

**Affiliations:** 1 Department of pediatric and child health Nursing, College of Health sciences, Debre Tabor University, Debre Tabor, Ethiopia; 2 Department of neonatal health Nursing, College of Health sciences, Debre Tabor University, Debre Tabor, Ethiopia; 3 Department of public health, College of Health sciences, Debre Tabor University, Debre Tabor, Ethiopia; 4 Department of midwifery, College of medicine and Health science, Wolaita sodo university, Wolaita, Ethiopia; 5 Department of Reproductive Health and Nutrition, School of Public Health, Woliata Sodo University, Woliata, Ethiopia; 6 Department of Epidemiology and Biostatistics, School of Public Health, Woliata Sodo University, Woliata, Ethiopia; 7 Department of Biochemistry, College of medicine and health sciences, Debre Tabor University, Debre Tabor, Ethiopia; 8 Unit of physiology, Department of Biomedical science, college of health science, Debre Tabor University, Debre Tabor, Ethiopia; 9 Department of Medical Biochemistry, College of Health Sciences, Debre Tabor University, Debre Tabor, Ethiopia; 10 Department of comprehensive Nursing, College of Health sciences, Wollo university, Wollo, Ethiopia; 11 Department of medical laboratory science, College of health sciences, Debre Tabor University, Debre Tabor, Ethiopia; 12 Department of comprehensive Nursing, College of Health sciences, Debre Tabor university, Debre Tabor, Ethiopia; 13 Department of Public Health, College of Health Sciences, Woldia University, Woldia, Ethiopia; 14 Department of Medical Biochemistry, College of Medicine and Health Sciences, University of Gondar, Gondar, Ethiopia; 15 Department of Epidemiology and preventive Medicine, School of Public Health and Preventive Medicine, Faculty of Medicine, Nursing and Health Sciences, Monash University, Melbourne, Victoria, Australia, and Department of Maternal and neonatal health Nursing, College of Health Sciences, Debre Tabor University, Debre Tabor, Ethiopia; 16 Department of comprehensive Nursing, College of Health sciences, Debre Berhan university, Debre Berhan, Ethiopia

**Keywords:** Children with cerebral palsy, Malnutrition, Sub-Saharan Africa, Molecular medicine, Neurology, Risk factors

## Abstract

Malnutrition is more prevalent among children with cerebral palsy and a major factor for child morbidity and mortality in children with different co-morbidity, especially in Sub-Saharan Africa: The main aim of this systematic review and meta-analysis was to estimate the burden of malnutrition among children with cerebral palsy in Sub-Saharan Africa. We searched PubMed, Web of Science, Google Scholar, Research Gate, and institutional repositories for papers that reported the proportion of malnutrition among children with cerebral palsy that were published between December 2010 and September 2023. Data were retrieved using the standardized JBI data extraction checklist through Microsoft Excel, and then exported to STATA 17 for further analysis. DerSimonian and Laird’s estimator was used to calculate the pooled effect size in the random-effects model. Statistics such as the Cochran Q test and I2 test were employed to measure heterogeneity. Egger's test and the funnel plot were used to look for publication bias. This systematic review and meta-analysis used 16 studies from Sub-Saharan Africa to estimate the proportion of malnutrition among 2,120 children with cerebral palsy. The pooled proportion of malnutrition among children with cerebral palsy in Sub-Saharan Africa by using random-effects model analysis was found to be 59.7% (95% CI; 49.8–69.6). The proportion of malnutrition was also estimated by sample sizes categorized as ≤ 120 and > 120, and the proportion of malnutrition was found to be 54.0 (95% CI: 44.7–63.3) and 64.5 (95% CI: 50.5–78.5). Moreover, the proportion of malnutrition was estimated by accounting for the difference in the year of publication. In this regard, the study classified before ≤ 2017 and > 2017, and the proportion of malnutrition was found to be 53.7 (95% CI: 38.0–69.3) and 62.5 (95% CI: 49.7–75.3) in Sub-Saharan Africa respectively. Malnutrition among children with cerebral in Sub-Saharan Africa was found to be very high. Hence, enhancing and developing strategic guidelines for malnutrition screening, prevention, and nutritional support are crucial among children with cerebral palsy. Furthermore, systematic review, randomized control trials, and qualitative studies are recommended to understand the burden more among children with cerebral palsy in the continent.

## Introduction

Cerebral palsy is a neurological disorder cause a lifelong physical disability^1^. With an estimated incidence of 17 million persons globally, cerebral palsy affects around 1 in 500 newborns^2^.

Children with cerebral palsy have a higher risk of developing malnutrition than other children because CP affects the muscles and movements involved in chewing, swallowing, and feeding^3,4^. It is a double burden for children with cerebral palsy to develop malnutrition since the problems are worsening each other and add challenges^5,6^.

The prevalence of malnutrition among children with cerebral palsy was 7.9–71.46%^5,7^, and malnutrition is more prevalent among children with cerebral palsy and a major factor for child morbidity and mortality in children with different co-morbidity, especially in Sub-Saharan Africa^8^.

Children with cerebral palsy are more susceptible to both the long- and short-term effects of malnutrition. Malnutrition lowers quality of life and social engagement, raises susceptibility to illness, and health care utilization, and lowers chances of survival^5,6,9^.

Therefore, malnutrition prevention, management, and nutritional support are very crucial and the stakeholders, government, and non-governmental organizations should be included in planning and implementing a package for children with cerebral palsy^5,10,11^.

Although some research has been done on the prevalence of malnutrition among children with cerebral palsy in Sub-Saharan Africa in the past, the combined frequency is not well understood. This systematic review and meta-analysis aimed to determine the pooled prevalence of malnutrition among children with cerebral palsy in Sub-Saharan Africa. This study may also draw new evidence from different heterogeneity of studies. Hence, this research is of paramount importance in providing evidence to policymakers, researchers, and clinicians to recognize the burden and can help the development of strategic guidelines, and enhance clinical decision-making and monitoring by integrating the recommendations of the study.

## Methods

### Data sources and search strategies

#### Review question

The review question of this systematic review and meta-analysis is: What is the burden of malnutrition among children with cerebral palsy in Sub-Saharan Africa?

#### Study selection

The Preferred Reporting Items for Systematic Reviews and Meta-Analyses (“PRISMA checklist” was used in the formulation of the systematic review methodology^12^. A registration request for the review methodology has been made to the PROSPERO database.

Articles were evaluated based on the inclusion and exclusion criteria from the perspective of the outcome variable (i.e., are they reporting the prevalence or epidemiology of malnutrition among children with cerebral palsy or not).

#### Search strategy

We searched PubMed, Google Scholar, Web Science, Cochrane databases, and other source that were conducted on malnutrition among children with cerebral palsy, and were published between January 2010 and September 2023. Since research involves constantly testing the hypotheses, staying updated with the latest publications will help us estimate the recent and updated burden of the problem.

The search was conducted using the following keywords: (Prevalence OR proportion OR Burden OR magnitude) AND (Children [MeSH Terms] OR Child OR infant OR Kids) AND (Cerebral palsy [MeSH Terms] OR Disability OR Impairment OR Special needs) AND (Sub-Saharan Africa) OR developing country OR resource-limited setting. The search terms were used separately and in combination using Boolean operators like “OR” or “AND”. The literature search conducted from April 01 to 17, 2023 by authors includes ESC, MAM, MAS, and SSC. All papers published until April 17, 2023, were included in this review.

### Eligibility criteria

#### Inclusion criteria

- *Language*: Articles published in English.
- *Study area*: Only studies conducted in Sub-Saharan Africa were eligible for this study.
- *Study design*: Observational studies (case-control, cohort, and cross-sectional study) that report the prevalence of malnutrition among children with cerebral palsy.
- *Publication year*: Reports made from January 2010 onwards.
- *Publication condition*: Peer-reviewed journal articles as well as articles from the university website.
- *Outcome of interests*: Studies reported the prevalence of malnutrition among children with cerebral palsy in Sub-Saharan Africa was considered.

#### Exclusion criteria

Due to the inability to evaluate the quality of publications without access to the complete text and to estimate the outcome variable, articles that were not fully accessed were excluded. Furthermore, studies that did not distinguish between children with cerebral palsy and those with other special needs/disability were excluded from the study.

### Outcome measurement among children with cerebral palsy

There is one key outcome variable in this systematic review, which is the prevalence of malnutrition among children with cerebral palsy. The prevalence of malnutrition among children with cerebral palsy was calculated by multiplying the total number of children with cerebral palsy who had malnutrition by 100 and dividing that number by the total number of children with cerebral palsy.

### Study selection and data extraction

All Studies reported the prevalence of malnutrition among children with cerebral palsy in Sub-Saharan Africa were transferred to Zotero software, and duplicate articles were removed. The pertinent information, including the names of the authors, the study's design, sample size, study area, and the number of cases/proportions of malnutrition were independently extracted by four authors (ESC, NM, FDB, and GBM). Similar to the preceding step, disagreements were discussed during a consensus meeting with other reviewers (MAM, MAS, ECA, MMG, KDT, SSC, and YSK) for the final decision on which studies to include in the meta-analysis and systematic review.

### Quality assessment

We evaluated the included studies' quality using the Joanna Briggs Institute Critical Appraisal instrument for use in JBI Systematic Reviews (JBI-MAStARI)^13^. For the cross-sectional, case–control, and cohort investigations, various items were used in the scale. This systematic review and meta-analysis study included five writers (TAD, WAB, AK, AMD, and MMA) who examined the included articles.

### Statistical analysis

The authors verified the accuracy of the retrieved data in a Microsoft Excel sheet before exporting it into STATA 17 for further analysis. The STATA version 17 was used for all analyses in this study.

The degree of heterogeneity was evaluated using Cochrane Q-statistics and an *I*^2^ statistic with a *p*-value (< 0.05)^14,15^. The test for heterogeneity was high (75%) or moderate (50–74%)^16^. Using a random-effects model, the pooled prevalence of malnutrition among children with cerebral palsy was estimated.

Once we had determined that there was moderate or higher heterogeneity, we also looked into the cause of the heterogeneity using subgroup analysis and meta-regression^17^. Predetermined subgroups were taken into account for this, including the study sample size, study design, publication year, and study conditions.

The publication bias was estimated through a funnel plot and Egger test^18^ and sensitivity analysis was piloted to examine the effect of a single study on the overall estimation.

## Results

### Search result

**Figure 1:**
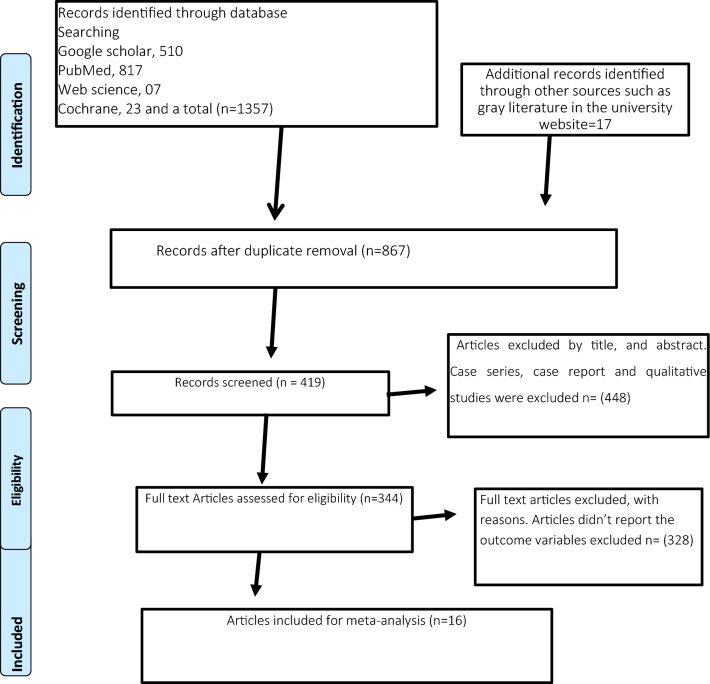
The PRISMA flow diagram of searching process.

We found 1374 studies from PubMed, Google Scholar, Web Science, Cochrane databases, and other source. There were 507 studies were excluded due to duplication. Of the remaining 867 studies, 523 studies were excluded through title and abstract screening. From a total of 344 full-text articles, 328 were excluded due to the outcome variable wasn’t reported. A total of 16 studies were finally included from 09 Sub-Saharan Africa such as Nigeria, Ghana, Uganda, Kenya, Sudan, Cameroon, Tanzania, Botswana, and Zambia (Fig. 1).

### Study characteristics and participants

This systematic review and meta-analysis examined 16 studies conducted in Sub-Saharan Africa to determine the prevalence of malnutrition among 2,120 children diagnosed with cerebral palsy. The review included a total of 16 cross-sectional studies with study design^19–34^.

Out of sixteen studies in this review, five were from Nigeria^19,21,23,25,26^,, three were from Ghana), two studies were from Uganda^22,28^, and the remaining seven studies were from Kenya^30^, Sudan^20^, Cameroon^29^, Tanzania^33^, Botswana^27^, and Zambia^31^.

The review found that the highest proportion of malnutrition among children with cerebral palsy was 90%^22^, while the lowest proportion was 37.3%^23^. The sample sizes varied significantly, with the largest study including 388 participants from Nigeria^25^, and the smallest study including only 16 participants from Zambia^31^ (Table 1).

**Table 1:** Distribution of malnutrition among children with cerebral palsy in Sub-Saharan Africa.

First author/year	Country	Study design	Sample size	Cases	Malnutrition (%)	Quality status
Adamu et al 2018	Nigeria	Cross sectional	150	129	86	Low risk
Ali et al. 2022	Sudan	Cross sectional	90	53	58.9	Low risk
Badaru et al. 2023	Nigeria	Cross sectional	146	95	65.1	Low risk
Bambi et al. 2021	Uganda	Cross sectional	224	202	90	High risk
Chidomere et al. 2023	Nigeria	Cross sectional	169	63	37.3	Low risk
Donkor et al. 2019	Ghana	Cross sectional	34	17	50	Low risk
Duke et al 2021	Nigeria	Cross sectional	388	198	51	Low risk
Iloeje et al. 2017	Nigeria	Cross sectional	100	37	38.1	Low risk
Johnson et al. 2017	Botswana	Cross sectional	61	26	43	Low risk
Kakooza- et al. 2015	Uganda	Cross sectional	135	57	42	Low risk
Kana et al. 2022	Cameroon	Cross sectional	88	36	40.9	Low risk
Koriata et al. 2012	Kenya	Cross sectional	140	98	70.3	Low risk
Nawa et al. 2022	Zambia	Cross sectional	16	13	80.5	Low risk
Polack et al. 2018	Ghana	Cross sectional	76	49	65	Low risk
Sissya et al. 2010	Tanzania	Cross sectional	239	175	73.1	Low risk
Zuurmond et al. 2018	Ghana	Cross sectional	64	40	63.1	Low risk

### Meta-analysis

**Figure 2:**
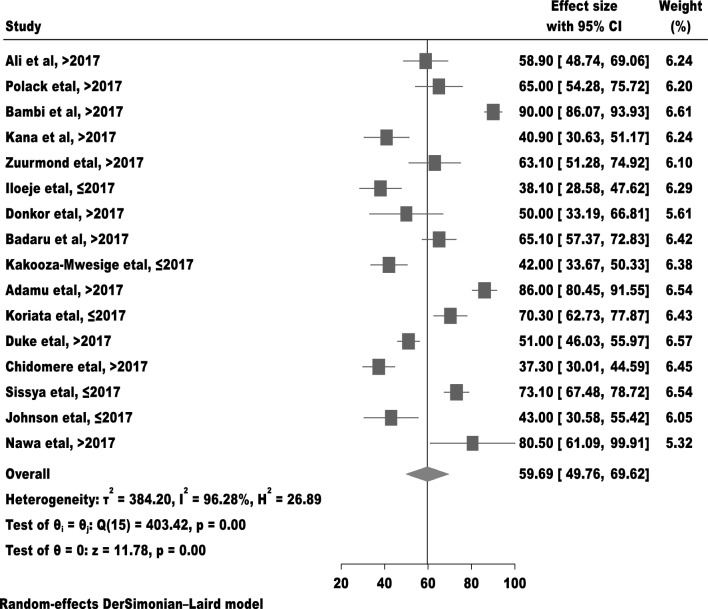
Forest plot to test of malnutrition estimation among children with cerebral palsy in Sub-Saharan Africa.

The pooled proportion of malnutrition among children with cerebral palsy in Sub-Saharan Africa by using random-effects model analysis was found to be 59.7% (95%CI; 49.8–69.6) and showed in (Fig. 2).

### Estimation of malnutrition burden by sub group analysis among children with cerebral palsy

**Figure 3:**
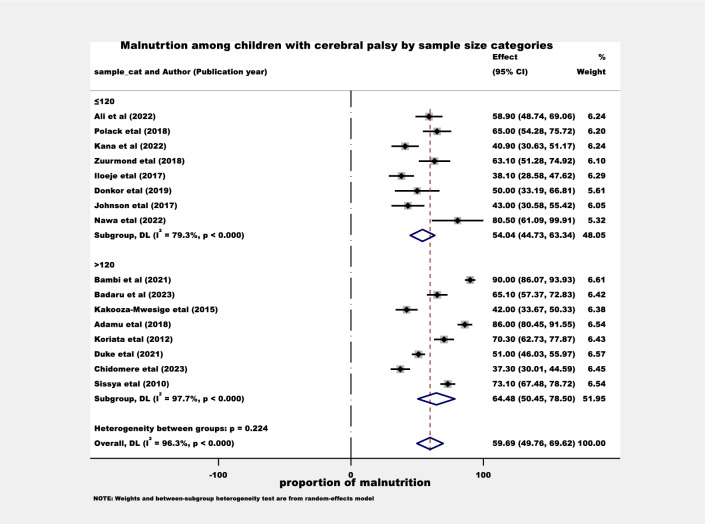
The proportion of malnutrition estimation among children with cerebral palsy by sample sizes categories.

**Figure 4:**
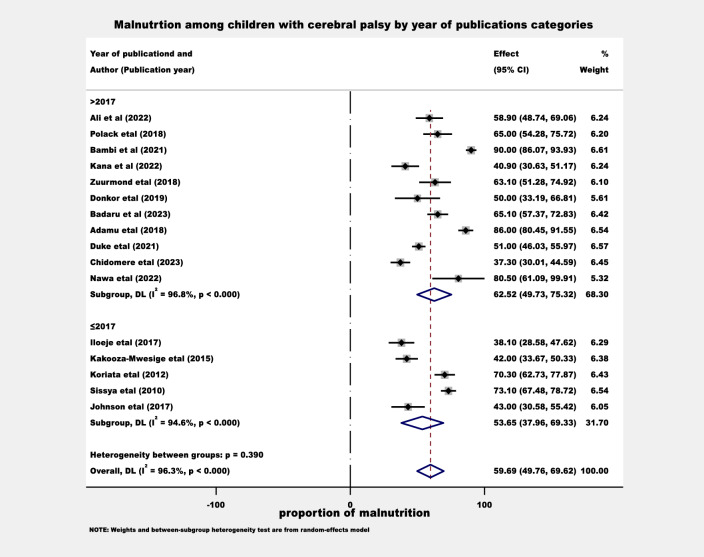
The proportion of malnutrition estimation among children with cerebral palsy by year of publications categories.

The proportion of malnutrition was also estimated by considering the sample size difference. The sample sizes were categorized as ≤ 120 and > 120, and the proportion of malnutrition was found to be 54.0 (95% CI: 44.7–63.3) and 64.5 (95% CI: 50.5–78.5) in Sub-Saharan Africa respectively (Fig. 3). Moreover, the proportion of malnutrition was estimated by accounting for the difference in the year of publication. In this regard, the study classified before ≤ 2017 and > 2017, and the proportion of malnutrition was found to be 53.7 (95% CI: 38.0–69.3) and 62.5 (95% CI: 49.7–75.3) in Sub-Saharan Africa respectively (Fig. 4).

### Publication bias

**Figure 5:**
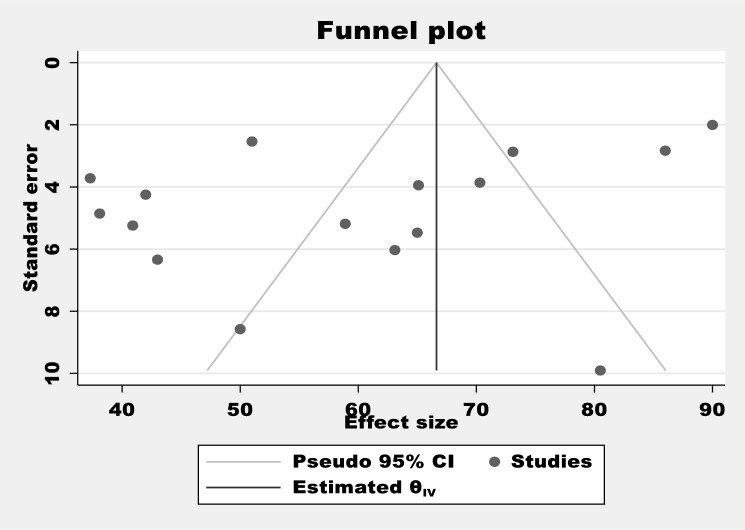
Funnel plot tests for the proportion of malnutrition among children with cerebral palsy in Sub-Saharan Africa.

**Figure 6:**
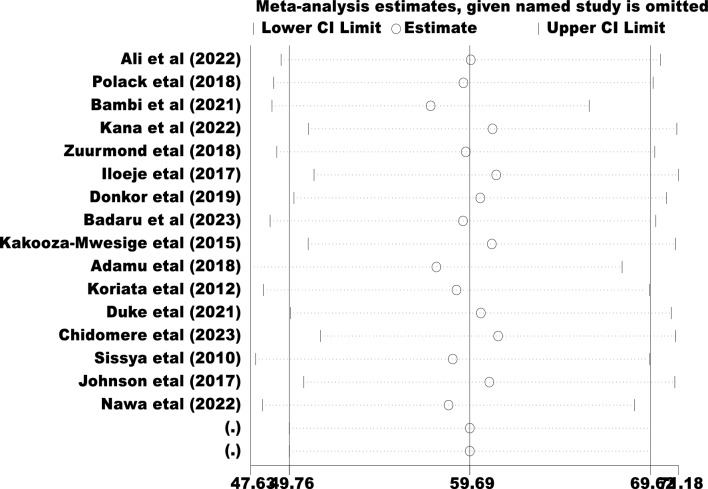
The sensitivity test for the proportion of malnutrition among children with cerebral palsy in Sub-Saharan Africa.

Egger's regression test value showed that there is a statistically significant publication bias in this study (*p* < 0.044) (Table 2). Besides, a funnel plot showed an asymmetrical distribution which indicated the presence of publication bias (Fig. 5)). Moreover, the results of sensitivity analyses using the random effect model suggested were estimated (Fig. 6).

**Table 2:** Egger's test of the study of malnutrition among children with cerebral palsy in Sub-Saharan Africa.

Std_Eff Coefficient		SE	T	*P* > t	[95% CI]	
Slope	89.32553	11.1347	8.02	0.000	65.44397	113.2071
Bias	−6.563965	2.965408	−2.21	0.044	−12.92413	−0.2037975
Test of H0: no small-study effects *P* = 0.044, Root MSE = 4.62, Number of Studies = 16

## Discussion

This systematic review and meta-analysis study provides representative data on the estimation of malnutrition among children with CP in Sub-Saharan Africa.

The study suggests that an estimated pooled malnutrition burden among children with cerebral in Sub-Saharan Africa was found to be 57.4% (95% CI; 46.7–68.2).

The finding of this review, the proportion of malnutrition among children with CP is higher than the study conducted in Saudi Arabia^11^, U.S.A^7^, Argentine^10^, Bangladesh^35^, Nepal^36^, Turkey^37^, Vietnam^38^, and systematic review and meta-analysis Asia 40%^39^.

This difference might be due to the limited healthcare services, healthcare providers, infrastructure, and limited social support for the optimization of health among children with cerebral palsy by preventing, controlling, and managing malnutrition in Sub-Saharan Africa as compared to other continents^40,41^.

Moreover, food insecurity, overpopulation, low educational status, and wars can contribute to malnutrition is too high, particularly in resource-limited settings and vulnerable groups such as children with cerebral play in Sub-Saharan Africa^40–43^.

However, the proportion of malnutrition is lower than in the study conducted in Colombia^9^, and a Systematic Review and Meta-Analysis Arabic speaking countries^5^. This difference might be due to the reference standards cut-off point to diagnosis malnutrition in Colombian and Arabic-speaking countries were included mild, modern, and severe classifications, whereas this study included moderate and severe to declare malnutrition only among children with cerebral palsy^5,9^.

The finding of this review will inform the burden of malnutrition among children with cerebral palsy in Sub-Saharan Africa for the planners, policymakers, healthcare providers, government and non-governmental organizations, and researchers’ up-to-date data. Additionally, this finding shows the impact of malnutrition in children with cerebral, so prevention and management through the integration of the recommendations to develop guidelines and improve the quality of service is very crucial for children with cerebral palsy in the continent.

The subgroup analysis conducted in this review aimed to estimate the proportion of malnutrition among children with cerebral palsy in various categories within the Sub-Saharan Africa region. One aspect that was examined was the difference in sample sizes, with participants categorized as ≤ 120 and > 120. The estimated proportions of malnutrition were found to be 54.0% (95% CI: 44.7–63.3) and 64.5% (95% CI: 50.5–78.5), respectively. Another factor considered was the variation in the year of publication, with studies classified as published before ≤ 2017 and > 2017. The estimated proportions of malnutrition were found to be 53.7% (95% CI: 38.0–69.3) and 62.5% (95% CI: 49.7–75.3), respectively. Taking into account the significance of a large sample size for accuracy, it is conceivable that the proportion of malnutrition among children with cerebral palsy in Sub-Saharan Africa might be higher than the findings of this study suggest. Moreover, recent studies focusing on this population in the region have shown that, there is an increment in the prevalence of malnutrition among children with cerebral palsy. It is crucial to take these subgroup analysis findings into account to avoid misleading conclusions influenced by confounding factors^44^.

The subgroup analysis findings emphasize the urgent requirement for interventions and targeted strategies to tackle the rising prevalence of malnutrition among children with cerebral palsy.

### Limitations of the study

This systematic review and meta-analysis have some limitations. Firstly, the study did not assess the specific causes of malnutrition among children with cerebral palsy. Secondly, only quantitative observational studies published in English were included in the analysis, potentially excluding relevant studies in other languages. Thirdly, studies that did not differentiate between children with cerebral palsy and those with other disabilities were not considered in the analysis.

## Conclusion

Malnutrition among children with cerebral in Sub-Saharan Africa was found to be very high. Hence, enhancing and developing strategic guidelines for malnutrition screening, prevention, and nutritional support are crucial among children with cerebral palsy. Furthermore, systematic review, randomized control trials, and qualitative studies are recommended to understand the burden more among children with cerebral palsy in the continent.

### Supplementary Information

Supplementary Information.

## Data Availability

All data generated or analyzed during this study are included in this published article.

## Abbreviation

CP: Cerebral palsy
